# Efficacy of Dienogest in Adolescent Endometriosis: A Narrative Review

**DOI:** 10.7759/cureus.36729

**Published:** 2023-03-27

**Authors:** Surekha Tayade, Sangeeta Rai, Hrishikesh Pai, Madhuri Patel, Nidhi Makhija

**Affiliations:** 1 Obstetrics and Gynaecology, Jawaharlal Nehru Medical College, Datta Meghe Institute of Medical Sciences, Wardha, IND; 2 Obstetrics and Gynaecology, Institute of Medical Sciences, Hindu University, Banaras, IND; 3 Obstetrics and Gynaecology, Federation of Obstetrics and Gynecological Societies of India, Mumbai, IND

**Keywords:** endometriosis, severe dysmenorrhea, pelvic pain, dienogest, adolescents

## Abstract

Teenage endometriosis is seen as a chronic condition that can worsen if untreated. Treatment objectives include relief of symptoms, prevention of disease development, and preservation of future fertility. In many regions, dienogest (DNG), an oral progestin, has emerged as a key treatment in suppressing endometriosis. The usage of DNG for endometriosis in adolescents was researched in papers published between 2015 and 2022 using PubMed and Google Scholar. A thorough search of all identified studies' reference lists and previously published literature reviews was carried out. The study's nature and geographic scope were not restricted. After reviewing these publications, the authors decided on which ones were the most pertinent in light of their personal experiences. The final study consisted of 14 studies that satisfied inclusion requirements. The trials showed that taking DNG 2 mg daily efficiently lowers endometriotic lesions, eases painful endometriosis symptoms, and improves quality-of-life indicators. In most of these investigations, DNG was shown to be safe and tolerated, with predictable and moderate side effects, good patient compliance rates, and low withdrawal rates. Although endometrioma did not enlarge while receiving treatment, significant regression was not typical. Overall, the studies found that DNG is safe and effective in reducing symptoms of endometriosis in adolescents.

## Introduction and background

Endometriosis is a chronic condition that primarily affects reproductive-age women. Endometriotic lesions that develop outside the uterus are generally accompanied by severe pain and heaviness before or during menstruation and infertility [1]. Adolescent years are when endometriosis symptoms commonly first appear in women, but diagnosis is frequently delayed.

Endometriosis has no cure. The primary objective of endometriosis treatment is the control of symptoms. The goals of management include minimising endometriotic lesions, treating pain, and enhancing the quality of life [2]. Several medications currently used to treat endometriosis have weak clinical evidence supporting their safety and efficacy, and recurrence after surgical intervention is common. Progestin is suggested as the first-line hormonal treatment for pain caused by endometriosis [3].

Dienogest (DNG), an oral progestin, has been a popular treatment choice for the suppression of endometriosis in many parts of the world. In 2009, the European Commission first approved DNG, a twice-daily progestin of the fourth generation, for the treatment of endometriosis [4]. When regularly taken, DNG has local antiproliferative and anti-inflammatory effects on endometriotic lesions and decreases systemic gonadotropin secretion. The progesterone receptor is where DNG binds. DNG differs from other progestins in its class due to these features [3].

Because endometriosis is chronic, successful treatment must strike a compromise between clinical effectiveness and symptom alleviation and an appropriate long-term safety profile [5]. As a result, the current paper provides a narrative review of the research in favour of DNG's use in the long-term management of endometriosis along with suggestions for clinical practitioners to take into account regarding its effectiveness, safety profile, and use in the treatment of adolescent endometriosis.

## Review

Methodology

The usage of DNG for endometriosis in adolescents was researched in papers published between 2015 and 2022 using PubMed and Google Scholar. Additionally, a thorough search of all identified studies' reference lists and previously published literature reviews was conducted. Endometriosis, adolescents, and DNG were all searched as a combination of terms and keywords. To both limit and enlarge the search, truncation and Boolean operators were used.

**Figure 1:**
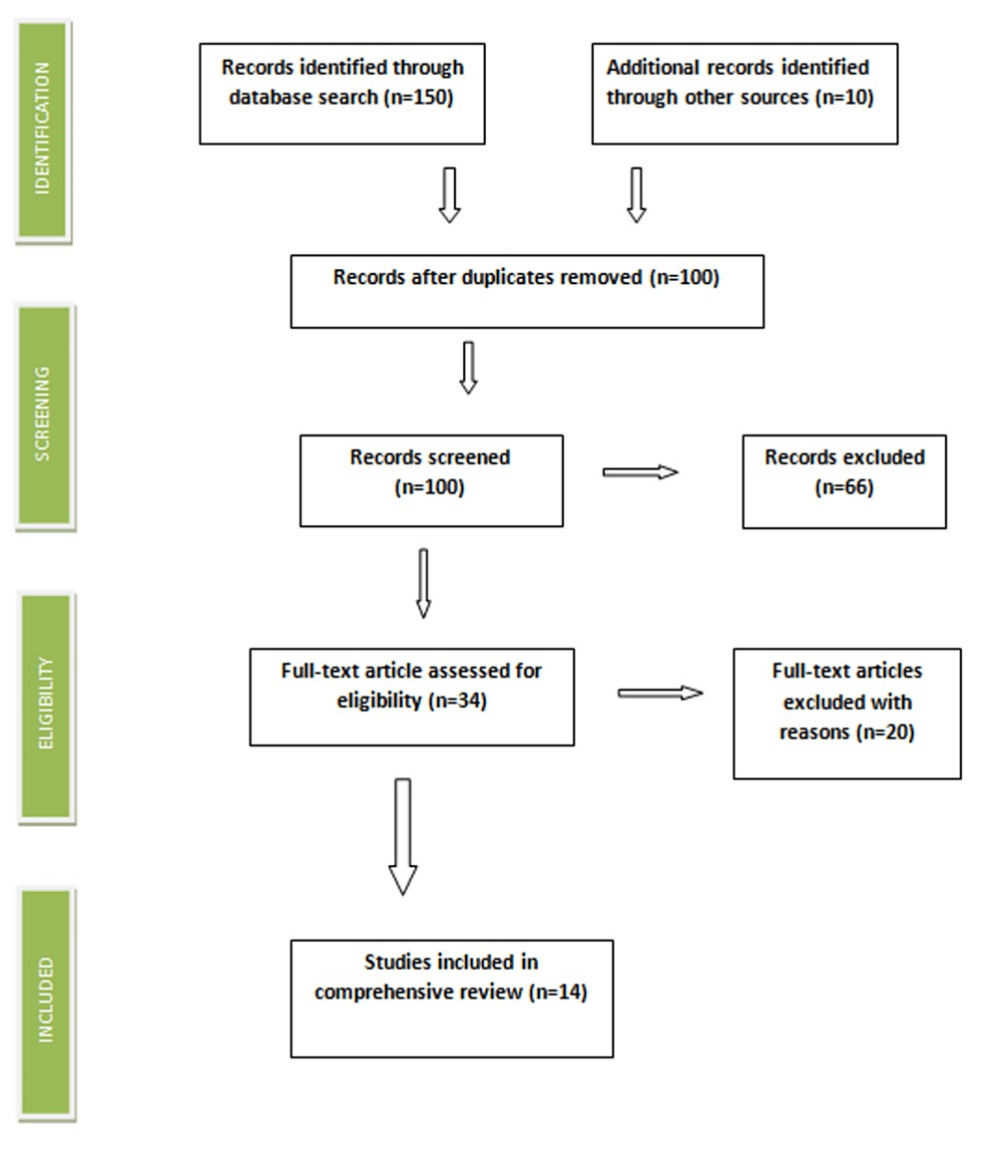
PRISMA flowchart

The eligibility criteria were that studies were included if one of their main aim(s) was to assess the role/efficacy/safety of DNG in treating endometriosis with study subjects either entirely or partially adolescents. No restriction was made on the type and geographical region of the study. Through the use of title and abstract relevancy, all discovered studies were screened in accordance with eligibility requirements. The authors studied the entire texts of studies that they thought would fit the criteria for this review. After reviewing these studies, the authors selected those that, in light of their personal experiences, they felt were the most pertinent. Finally, this review included 14 studies on DNG in adolescent endometriosis. The PRISMA chart for our assessment is depicted below (Figure 1).

Safety of DNG

In order to determine the safety and effectiveness of DNG 2 mg in teenagers with suspected endometriosis, Ebert et al. [6] carried out a 52-week, open-label, single-arm study in 21 research centres across six different European countries. DNG 2 mg once daily was administered to 111 adolescents between the ages of 12 and less than 18 who had endometriosis that was either clinically suspected or proven by laparoscopy. They used a visual analogue scale (VAS) to assess changes in endometriosis-related pain and dual-energy X-ray absorptiometry to measure relative changes in bone mineral density (BMD) of the lumbar spine (L2-L4). One hundred and twenty people were screened for the experiment, and 111 of them finished it. The mean VAS score of endometriosis-related pain was reduced significantly from 64.3 (SD, 19.1) mm at baseline to 9.0 (SD, 13.9) mm after the 48th week of treatment.

In teenagers with probable endometriosis, DNG 2 mg for 52 weeks was linked to a reduction of lumbar BMD, which was then partially reversed following medication termination. The findings demonstrate that progestin dramatically improved pain associated with endometriosis while simultaneously reducing lumbar BMD. The study emphasises the requirement for tailored care in this demographic, taking into account a person's risk factors for osteoporosis as well as the treatment's anticipated efficacy for pain caused by endometriosis. Adolescence is a pivotal time for bone accretion. DNG had no effect on BMD, according to the research by Yu et al [7]. After three months of DNG 1 mg/day in young women with dysmenorrhea and irregular menstruation, aged 10-24 years, Ota et al. [8] conducted a retrospective cohort study to assess bone turnover. The levels of the bone metabolism markers TRACP-5b and BAP, as well as the levels of gonadotropins, were measured before and after three months of low-dose DNG 1 mg/day treatment. They discovered that giving young women DNG at a dose of 1 mg per day for three months had no appreciable impact on bone turnover during the bone-growth phase. DNG was found to be generally well tolerated in clinical trials [9-12]. Here is an understanding of the biological factors of different progesterones used in endometriosis [13] (Table 1).

**Table 1:** Progesterones used in endometriosis MPA, medroxyprogesterone acetate; DNG, dienogest; RANTES, regulated on activation, normal T-cell expressed, and secreted; Bcl-2, B cell lymphoma/leukaemia-2

Drugs	Biological effects
MPA	Plasminogen activator inhibitor production stimulation to prevent angiogenesis in vitro, nuclear factor K-B transcription activity inhibition in vitro, and progesterone receptor-mediated down-regulation of endometrial RANTES gene transcription in vitro
DNG	Induction of prolactin production by human endometrial stromal cells in vitro, normalisation of in vivo peritoneal fluid cell count, interleukin-1ß production by peritoneal macrophages is reduced in vivo, natural killer activity of peritoneal fluid cells increases in living things, inhibition of protein kinase C activity in vitro in rat endometrial cells, and in vivo inhibition of angiogenesis human endometrial stromal cell growth
Oral contraceptive (Desogestrel 0.15 mg + ethinyl estradiol 0.03 mg)	Decreased Ki-67 protein expression in the eutopic endometrium of endometriosis-affected women, increased endometrial apoptosis in endometriosis-afflicted women, Bcl-2 expression is downregulated, and Bax is upregulated in the eutopic endometrium of endometriosis-affected women

Efficacy of DNG

Malik and Mann [9] carried out a short-term single-centre study on the “Role of Dienogest in Endometriosis in Young Women” among 56 patients between the ages of 15 and 35 years who complained of pain and were laparoscopically or by imaging examinations identified as having endometriosis. DNG 2 mg once daily was administered to the patients, and the treatment's results were seen over the course of three months by an improvement in pain score. DNG's impact on endometrioma size was also seen. Patients were checked on after one and three months. They discovered that DNG significantly reduces discomfort [14]. Although endometrioma did not enlarge throughout treatment, it was shown that significant regression was rare.

A prospective, non-interventional study was carried out by Techatraisak et al. [15] in six Asian nations to evaluate the health-related quality of life (HRQoL) of patients receiving DNG in a practical context. The inclusion criteria for the trial comprised all women under the age of 18 years who had endometriosis, experienced endometriosis-associated pelvic pain (EAPP), and had started using DNG. Most patients and medical professionals rated DNG favourably. More than 80% of patients reported an improvement in their symptoms. Women who have had endometriosis surgically removed or clinically identified report better HRQoL and EAPP in the real world.

**Figure 2:**
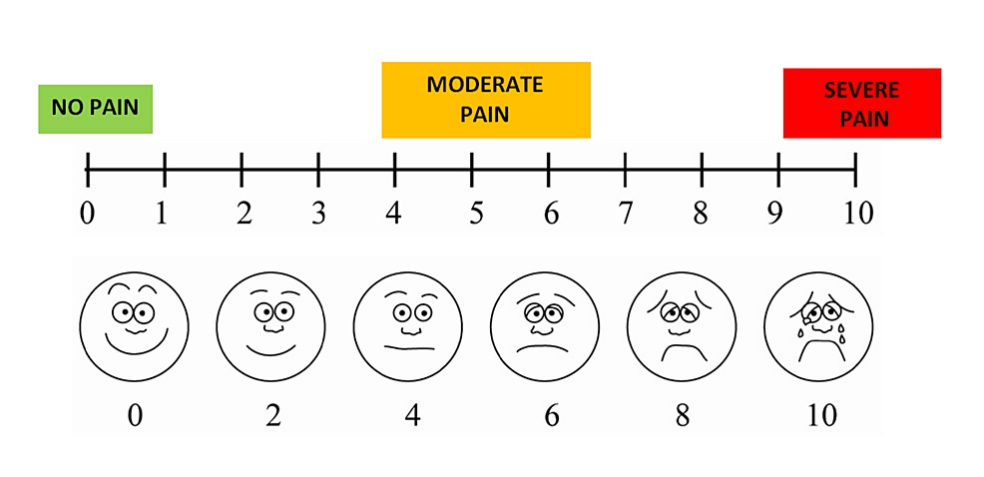
Visual analogue scale (VAS)

Yu et al. [7] conducted open-label extension research to evaluate the efficiency and safety of DNG for an additional 28 weeks. Once the 24-week, placebo-controlled research was finished, the endometriosis-affected women who had been enrolled (n = 220) were included in this investigation. Their evaluations also proved that DNG's efficacy was maintained or increased after an additional 28 weeks of treatment. In the universities of Siena, Milan, Cagliari, Perugia, Busto Arsizio, Pisa, Padua, Palermo, Foggia, Roma, Pescara, and Catanzaro, 142 endometriosis patients who took DNG 2 mg/day for 90 days participated in a prospective observational multicentre experiment. Each patient's quality of life was evaluated both before and after therapy, and their pelvic discomfort was rated on VAS on a scale from 0 to 10 (Figure 2). The mean VAS for endometriosis-affected women had significantly decreased at the trial's end. Together with the mental index score values, the physical index also rose. Headaches, bleeding, sadness, breast pain, and acne were the most frequent adverse events (AEs) during the therapy phase; however, none of these AEs prompted removal from the study. Overall, they found that DNG improves endometriotic women's quality of life by being effective and well-tolerated.

Side effects of DNG

Menstrual irregularities were the side effect(s) that were reported most frequently [11]. Other side effects were a brief headache, bleeding, sadness, breast soreness, and acne [10]. According to Yu et al. [7], DNG initiation was associated with longer but fewer spotting/bleeding episodes (Table 2). As the medication continued, the frequency and severity of the bleeding gradually diminished. Few patients with treatment-emergent AEs, which were mostly mild or moderate, withdrew from the open-label study [7]. In their research, Techatraisak et al. reported that treatment-emergent adverse medication events include vaginal bleeding, metrorrhagia, and amenorrhea [15].

**Table 2:** Side effects of DNG DNG, dienogest

Side effects of DNG
Menstrual irregularities
Weight gain
Nausea
Breast discomfort
Lower abdominal pain
Epigastric pain
Acne
Behavioural changes
Headache
Flatulence

Discussion

According to Ebert et al., there was a partial recovery in the lumbar spine BMD after the 52-week therapy of teenage endometriosis with DNG 2 mg [6]. Compared to what was shown in the VISADO (VISanne study to assess safety in ADOlescents) trial, certain individuals may experience more significant lumbar spine or total BMD abnormalities. Due to the importance of bone accretion during adolescence, there is a need to weigh the risk-benefit ratio of DNG 2 mg in particular adolescent patients or any significant risk for osteoporosis.

In Chinese women with endometriosis, DNG 2 mg once daily is effective and safe for the long-term control of EAPP, according to Yu et al. [7], with consistent EAPP decreases and irregular bleeding throughout prolonged treatment. The results of this study's effectiveness and safety analyses were in line with those of earlier investigations in Caucasian patients.

Three months after DNG 1 mg/day treatment for dysmenorrhea in young women, including adolescent girls, Ota et al. [8] reported that there was no obvious difference in bone turnover. DNG 1 mg/day may soon play a significant role in treating young women's dysmenorrhea.

Thirty-eight (67.2%) out of the 56 patients reported experiencing pain relief between two and five days after starting DNG, according to Malik and Mann [9]. Forty-one out of the 56 patients had significant pain at enrollment, and only one (1.79%) reported severe pain at the conclusion of the first month on DNG. Three patients (5.3%) significantly reduced the size of their endometriotic cysts (by more than 50%) after taking DNG for one month. Significant pain relief (>30%) was reported in 41 out of 56 patients (73.2%) who had been receiving treatment for three months. At the end of three months, DNG treatment resulted in considerable cyst size reductions (>50%) in seven patients (12.5%). There were no harmful adverse effects discovered.

The mean SD of the VAS was 8.2 in endometriosis-affected women, but at the end of the experiment, it had dramatically decreased to 5.9 ± 2.6, according to Luisi et al. [10]. The mental index score values climbed from 39.0 ± 9.8 to 46.0 ± 9.1, similar to how the physical index increased from 39.6 ± 9.6 to 47.7 ± 8.5 (p < .001). Headache (30.8%), bleeding (29.4%), sadness (26.6%), breast discomfort (23.8%), and acne (2%) were the most frequent AEs during the therapy period. However, none of these AEs called for withdrawal from the study.

According to a study by Miao et al. [12], the median VAS score for group 1 was 80 mm before the injection of gonadotropin-releasing hormone agonist (GnRH-a) and 10, 10, 10, 20, and 20 mm, respectively, at 0, 6, 12, 18, and 24 months of DNG treatment. There was a reduction in the mean uterine volume from 262.9 to 104.7 ml after GnRH-a therapy, which then slowly climbed from 104.7 to 139.5 ml after 24 months of DNG therapy. All laboratory measurements fell within the expected range. DNG may be given as a maintenance treatment after the injection of GnRH-a has discontinued.

A prospective observational study was carried out by Vignali et al. [16] to examine whether DNG could identify significant changes in size and symptoms. Patients with pain complaints and at least one endometrioma identified by transvaginal ultrasonography (TVS) were included. Using the length × depth × width × 0.5233 formula and the VAS, they measured the volume of the endometrioma and the pain sensations, respectively. Every day, 2 mg of DNG was given. After six and 12 months of therapy, follow-up appointments were planned to evaluate patients' symptoms and the size of their endometriomas. Sixty patients were enrolled, and 60 of them finished the six-month course of treatment. After six months, the mean volume decreased by 66.71%. The 12-month treatment was successfully completed by 58 patients. After a year, the mean volume had decreased by 76.19%. Dysmenorrhea decreased by 74.05% after six months and by 96.55% after a year. Patients reported reductions in dyspareunia and chronic pelvic pain (CPP) of 42.71%, 48.91%, and 51.93%, respectively, after 6 and 12 months. DNG causes a statistically significant decrease in the size and discomfort of endometriomas.

In a prospective study, Uludag et al. [11] looked at the effectiveness of DNG (2 mg/day) in the first, third, and sixth months of treatment for endometrioma. From the baseline to the six-month follow-up, the endometrioma's mean volume dramatically decreased. In the sixth month of treatment, the VAS score for pelvic discomfort also experienced a considerable decline.

According to Kizilkaya et al. [17], the mean endometrioma volume was considerably lower after therapy than it was before. Dysmenorrhea, dyspareunia, and persistent pelvic pain post-treatment VAS values all showed a significant reduction. The average scores for both physical and mental health improved dramatically. The size of the endometrioma, the amount of discomfort, and the quality of life in women with endometriosis were all considerably reduced by DNG treatment when given orally at doses of 2 mg/day for three months.

Ji et al. [18] conducted a cohort research in symptomatic females with uterine adenomyosis to evaluate the effectiveness and security of DNG and GnRH-a. They found that DNG effectively managed the dysmenorrhea symptoms in adenomyosis patients but was unable to manage anaemia or uterine shrinkage. GnRH-a was discovered to be more effective in treating anaemia and reducing uterine volume in patients with adenomyosis. Miao et al. conducted research on women with symptomatic adenomyosis [12]. Group 1 (maximum uterine dimension, 100.0 mm) started DNG four months after starting GnRH-a treatment in contrast to Group 2 (maximum uterine dimension, 100.0 mm), which started it immediately after stopping GnRH-a treatment. At the beginning of the treatment and then every six months after that, measurements of the uterine diameters and all of the women's baseline pain complaints were taken. They found that the average uterine volume continuously rose after GnRH-a therapy and two years of DNG medication. The mean uterine volume of 52 additional women with mild uterine enlargement who got DNG without first receiving GnRH-a after 24 months of treatment showed a small decrease (p > .05). Overall, they discovered that after GnRH-a administration is halted, DNG can be utilised as a maintenance therapy.

The size of the endometrioma cysts in the DNG group was dramatically decreased, according to Angioni et al. [19] and their research on the medicine DNG. A 75% volume decrease was achieved in the DNG group due to the mean cyst diameter decreasing from 52 mm at baseline to 32 mm after six months of therapy (p < .001).

According to Muzii et al. [20], medical treatment with DNG greatly decreases the diameter of endometriomas and the pain they cause, but the ovarian reserve appears to be conserved because of a considerable increase in antral follicle count (AFC) and a lack of a significant change in anti-Mullerian hormone (AMH). Angioni et al. [21] in their study likewise discovered that DNG medication significantly reduced the size of the nodules and swiftly relieved symptoms.

The table below gives a detailed summary of all the articles in the study (Table 3).

**Table 3:** Table depicting relevant articles in the study DNG, dienogest; AFC, antral follicle count, AMH, anti-Mullerian hormone; AEs, adverse events; GnRH-a, gonadotropin-releasing hormone agonist

Sr. No.	Study	Design	Sample size	Intervention	Duration	Comments
1.	Ebert et al., 2017 [6]	Open-label, single-arm study	111	DNG, 2 mg/day	52 weeks	Endometriosis-associated pain was substantially reduced during treatment, but It was discovered that DNG 2 mg was connected to a decline in lumbar BMD, which was followed by a partial recovery following treatment cessation
2.	Malik and Mann, 2021 [9]	Short-term single-centre study	56	DNG, 2 mg/day	3 months	DNG is a well-tolerated drug for endometriosis showing significant relief of pain with no major side effects. Although endometrioma did not enlarge throughout treatment, it was shown that significant regression was rare
3.	Techatraisak et al., 2022 [15]	Prospective, non-interventional study	887	DNG, 2 mg/day	24 months	From baseline to month 24, rates of normal bleeding decreased while rates of amenorrhea increased. Most patients and doctors expressed satisfaction with DNG. More than 80% of patients said their symptoms had improved. Drug-related treatment-emergent side effects, such as vaginal bleeding (10.4%), metrorrhagia (7.3%), and amenorrhea (6.4%), were experienced by 39.9% of patients
4.	Ota et al., 2021 [8]	Retrospective cohort study	321	DNG, 1 mg/day	3 months	In young women's bone-growth phase, DNG 1 mg/day treatment had no appreciable impact on bone turnover after three months
5.	Yu et al., 2018 [7]	Open-label extension study	220	DNG, 2 mg/day	28 weeks	Initiation of the DNG was related to longer but fewer spotting/bleeding episodes. As the medication was continued, the frequency and severity of bleeding gradually decreased. Treatment-emergent AEs, typically mild or moderate, resulted in discontinuation in the open-label study, and the DNG had no effect on BMD
6.	Luisi et al., 2015 [10]	Prospective observational multicentre study	142	DNG, 2 mg/day	90 days	The mean VAS in women with endometriosis significantly decreased at the end of the study. Mental index score values increased and the physical index increased. The most prevalent AEs during the treatment period were headaches, followed by bleeding, depression, breast soreness, and acne; however, none of these were persistent enough to cause study discontinuation
7.	Vignali et al., 2020 [16]	Prospective observational study	70	DNG, 2 mg/day	12 months	After a year, the mean volume had decreased by 76.19%. Dysmenorrhea decreased by 74.05% after six months and by 96.55% after a year. Patients reported reductions in dyspareunia and chronic pelvic pain of 42.71%, 48.91%, and 51.93%, respectively, after 6 and 12 months. DNG results in a statistically significant decrease in the size and discomfort of endometriomas
8.	Uludag et al., 2021 [11]	Prospective study	30	DNG, 2 mg/day	6 months	At six months into treatment, the VAS score for pelvic discomfort considerably decreased from 7.50 to 3.00 (p < .001). Inconsistencies in menstruation were the most frequent negative effects. The study's parameters for the lab remained constant. DNG was thought to have a favourable safety and tolerability profile and be effective for 6 months in reducing the size of endometriomas and the pain associated with endometriosis
9.	Kizilkaya et al., 2020 [17]	Prospective cohort study	37	DNG, 2 mg/day	3 months	The mean endometrioma volume was significantly reduced compared to the pre-treatment volume. Dysmenorrhea, dyspareunia, and persistent pelvic pain post-treatment VAS values all showed a significant reduction. The mean physical function score and mental health score significantly increased. They discovered that DNG treatment at a oral dose of 2 mg/day for 3 months significantly decreased endometrioma size, pain intensity, and quality of life in women with endometriosis
10.	Ji et al., 2022 [18]	Cohort study	127	The first group DNG 2 mg/day; second group received goserelin acetate (GS) (3.6 mg/4 weeks)	12 weeks	In individuals with adenomyosis, DNG efficiently reduces the symptoms of dysmenorrhea, but it is unable to relieve anaemia or shrink the size of the uterus
11.	Miao et al., 2022 [12]	Retrospective observational study	104	52 group 2 (maximum uterine dimension, 100.0 mm): DNG without prior GnRH-a treatment; 52 group 1 (maximum uterine dimension, 100.0 mm): DNG after 4 months of GnRH-a administration	24 months	After 24 months of DNG treatment, the mean uterine volume marginally dropped from 157.9 to 153.3 ml (p > .05). All laboratory measurements fell within the expected range. As a long-term treatment for symptomatic adenomyosis, DNG is efficient and well tolerated. It can also be used as maintenance therapy following the cessation of GnRH-a administration
12	Angioni et al., 2020 [19]	Prospective study	81	40 were given (DNG 2 mg/day); 41 were given ethinyl estradiol 30 mg [EE] with DNG 2 mg (DNG + EE)	6 months	The size of the endometrioma cysts was significantly reduced in the DNG group. The mean cyst diameter was 52 ± 22 mm at baseline and 32 ± 12 mm after six months of treatment (p < .001)
13.	Muzii et al., 2020 [20]	Prospective study	32	DNG, 2 mg/day	6 months	Medical treatment with DNG significantly reduced the endometrioma diameter and associated pain, whereas the ovarian reserve appears to be preserved, with a significant improvement of AFC and no significant change in AMH
14.	Angioni et al., 2015 [21]	Pilot study	6	DNG, 2 mg/day	12 months	Pain symptoms get better, and the nodule size gets smaller

## Conclusions

Overall, the studies found that DNG is safe, tolerable, and effective in reducing pain and in the treatment of endometriosis. DNG should be considered as an alternative for controlling symptoms related to endometriosis. The research also shows that DNG has brief and minor adverse effects; therefore, if patients are ready to accept the irregular bleeding and transient side effects that frequently accompany DNG, considering its effectiveness, then it can be utilised. It may be beneficial to talk to patients about potential side effects of DNG, such as headache, menstrual irregularities, weight gain, acne, and behavioural changes, and explain its safety and efficacy in the treatment of endometriosis when compared to other medications.
